# Host–Microbe Crosstalk in Sjögren’s Disease: Epithelial Type I Interferon Amplification, Loss of Short-Chain Fatty Acid Restraint, and the Effector-Phase Focus of Validated Therapy

**DOI:** 10.3390/ijms27209042

**Published:** 2026-10-12

**Authors:** Minjae Yu, Dahee Jo, Yeonhee Hwang, Kyudong Han

**Affiliations:** 1 Department of Bioconvergence Engineering, Dankook University, Yongin 16890, Republic of Korea; sscsmy12@gmail.com; 2 Center for Bio-Medical Engineering Core Facility, Dankook University, Cheonan 31116, Republic of Korea; daxhee0407@gmail.com (D.J.); hwang.yh.378@gmail.com (Y.H.); 3 Smart Animal Bio Institute, Dankook University, Cheonan 31116, Republic of Korea; 4 Department of Microbiology, College of Science & Technology, Dankook University, Cheonan 31116, Republic of Korea; 5 Global Chemical Convergence Research Institute, Dankook University, Cheonan 31116, Republic of Korea

**Keywords:** Sjögren’s disease, host-microbe interaction, type I interferon, short-chain fatty acids, salivary gland epithelium, oral and gut microbiome, multi-omics, tertiary lymphoid structure, mechanism-targeted therapy

## Abstract

Sjögren’s disease (SjD) is a systemic autoimmune disease of the exocrine glands, long framed as a lymphocyte-driven disorder in which immune cells destroy the target tissue. Genomic, epigenetic, microbiome, and single-cell and spatial studies have since shown the glandular epithelium and the microbial environment to be active participants rather than passive bystanders. This review asks whether host susceptibility and microbiome change can be joined into one continuous axis, following it from genetic and epigenetic susceptibility, through microbiome change read functionally rather than taxonomically, to the crosstalk that converts microbial signals into a host response and the cell states carrying it in human tissue. Two axes run through the whole sequence: the type I interferon (IFN) response and short-chain fatty acid (SCFA) metabolism. Read against them, every disease-modifying therapy with validated efficacy so far acts in the downstream B-cell effector axis, whereas the upstream IFN axis has yielded no established treatment. Because the microbial layer and the salivary gland transcriptome or proteome have never been measured in the same patients, the model proposed here remains a testable hypothesis rather than a completed causal chain. The aim is to specify that model, the falsification conditions it carries, and the study design its verification requires.

## 1. Introduction

Sjögren’s disease (SjD) is a systemic autoimmune disease characterized by chronic inflammation and functional decline of the exocrine glands. It was long understood as an immune-cell-centered disease in which lymphocytes infiltrate the glands and destroy the tissue, but genomic, epigenetic, microbiome, and single-cell omics studies have accumulated evidence that this view alone cannot account for. Throughout, this review refers to the disease as SjD, noting the original designation where a study enrolled only patients with primary Sjögren’s syndrome (pSS).

The clearest emblem of this change is the disease name. The 2023 International Rome consensus replaced Sjögren’s syndrome with Sjögren’s disease, redefined the standalone form as SjD and the form accompanying another connective-tissue disease as associated SjD, and removed the primary-versus-secondary distinction from clinical practice [1]. Unlike the term syndrome, SjD denotes a single disease entity defined by characteristic autoantibodies, salivary gland histopathology, and a particular pattern of systemic involvement, resetting the disease from a framework defined solely by immune-cell abnormality to one that also encompasses the target tissue and its microenvironment. Because an accompanying connective-tissue disease can still affect host genetic background, immune environment, and microbiome composition, it remains useful in pathophysiological research to analyze standalone and associated SjD separately. With the concept of autoimmune epithelitis, in which the salivary epithelium actively participates in antigen presentation and local immune responses, the target tissue came into focus as a participant [2], and SjD is now increasingly framed as a host-response disease in which immune cells, the target tissue, and the microbial environment operate together [3,4].

Current evidence comes largely from three sources. Genome-wide association and functional-annotation studies have linked the human leukocyte antigen (HLA) class II region and numerous non-HLA loci to SjD risk. Most regulate gene expression rather than alter protein sequence, converging on the type I interferon (IFN) response, B-cell activation, and nuclear factor kappa B (NF-κB) regulation [5]. Anti-Ro/SSA and anti-La/SSB autoantibodies and focal lymphocytic sialadenitis are central to diagnosis, and autoantibody subtype is associated with phenotype and prognosis [6]. The microbiome is the most recent axis, with several independent cohorts reporting oral and gut compositional change [7]. Each axis has accumulated substantial evidence, but attempts to join the three into one continuous pathophysiological flow have been limited.

**Figure 1:**
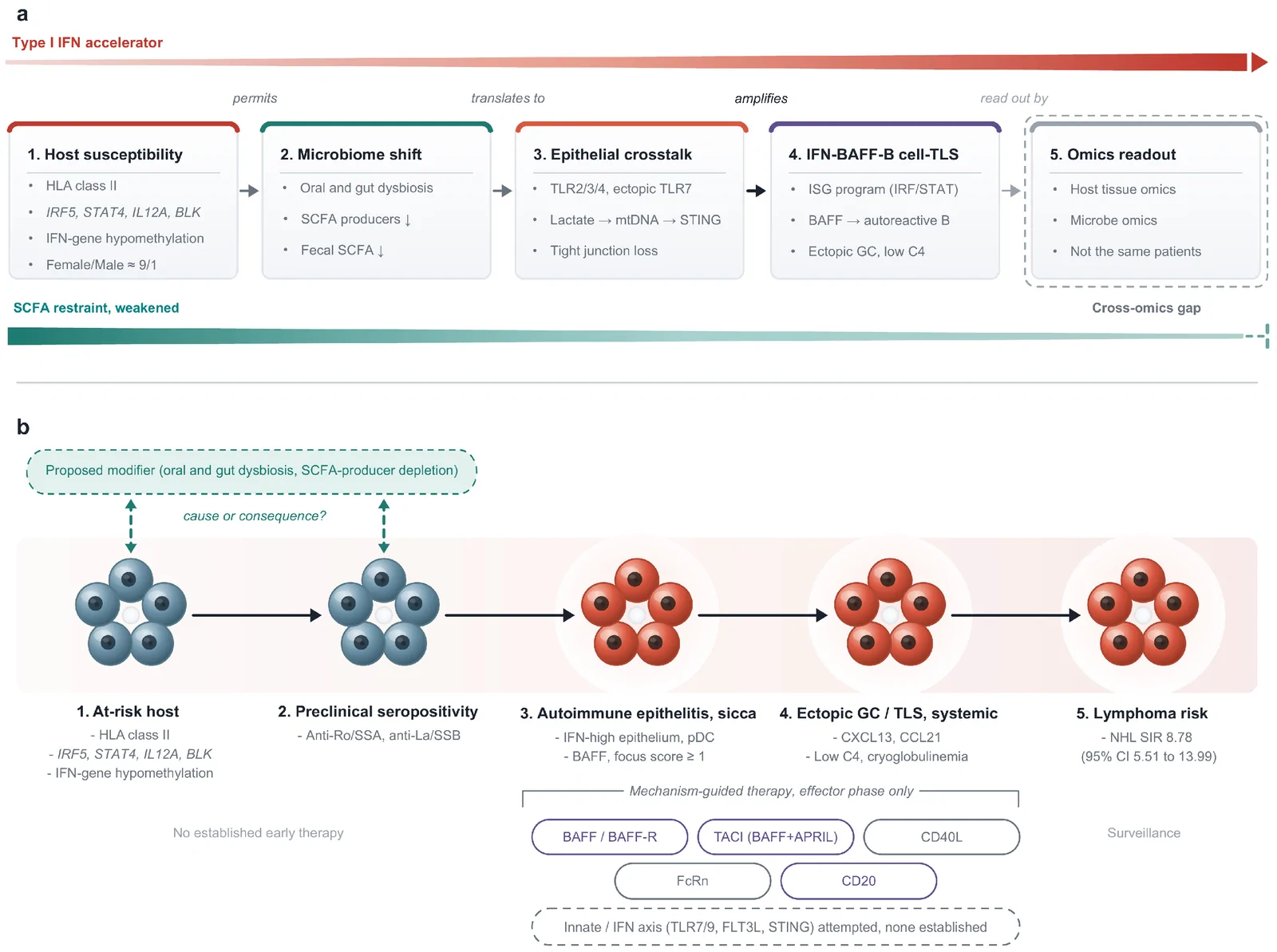
A type I interferon (IFN) accelerator and a weakened short-chain fatty acid (SCFA) brake linking host susceptibility to salivary gland inflammation in Sjögren’s disease (SjD). (**a**) Proposed host–microbe axis in five steps, from host genetic and epigenetic susceptibility to the omics readout, through the circuit formed by IFN, B-cell-activating factor (BAFF), B cells, and tertiary lymphoid structures (TLS). (**b**) Clinical continuum from an at-risk host to lymphoma risk, with the disease phase at which each target class has been tested. Dashed outlines mark proposed or unestablished elements. The upper red bar marks the type I IFN accelerator, widening as it amplifies, and the lower teal bar marks the SCFA restraint, fading into a broken T-bar as the brake is lost; downward arrows denote decrease in SjD relative to controls. In (**b**), acini turn from slate to red as the gland becomes IFN-high and inflamed, solid arrows indicate stage progression, purple pills mark agents acting directly on B cells, and bidirectional dashed arrows mark an undetermined direction. Abbreviations: APRIL, a proliferation-inducing ligand; BAFF-R, BAFF receptor; CD40L, CD40 ligand; CI, confidence interval; FcRn, neonatal Fc receptor; FLT3L, FMS-like tyrosine kinase 3 ligand; GC, germinal center; HLA, human leukocyte antigen; IRF, interferon regulatory factor; ISG, interferon-stimulated gene; mtDNA, mitochondrial DNA; NHL, non-Hodgkin lymphoma; pDC, plasmacytoid dendritic cell; SIR, standardized incidence ratio; STAT, signal transducer and activator of transcription; STING, stimulator of interferon genes; TACI, transmembrane activator and CAML interactor; TLR, Toll-like receptor.

This review therefore follows a single continuous host–microbe axis, asking which microbial change produces which gene- or protein-level change in which host cell type and how that response drives chronic inflammation. The question cannot be answered as a closed causal chain from current evidence. Based on a search of PubMed/MEDLINE and Europe PMC through 10 July 2026, it was determined that the microbial layer and the salivary gland transcriptome or proteome have never been measured together in the same patients. This narrative review therefore offers a testable hypothesis rather than a settled conclusion. It is a framework assembled across separate studies (Figure 1), carrying the falsification conditions and study designs its verification would require.

## 2. Genetic and Epigenetic Architecture of the Host Response

### 2.1. HLA and Non-HLA Susceptibility Loci

The most consistently reproduced genetic risk signal in SjD lies in the HLA class II region (Table 1). Centered on HLA-DR and HLA-DQ, it recurs across European and East Asian cohorts and connects directly to the first step of pathogenesis through presentation of Ro/SSA- and La/SSB-derived peptides to CD4^+^ T cells [5,8]. The stronger signal in anti-Ro/SSA-positive patients suggests that the region acts on both autoantigen recognition and autoantibody formation [9]. Normal salivary epithelium is not a professional antigen-presenting cell, yet increased HLA-DR expression has been reported in SjD salivary epithelium [10], so target-tissue epithelium exposed to inflammation may acquire an antigen-presentation-like state.

Non-HLA loci are scattered across the genome but converge on a few immune axes, which Table 1 sets out locus by locus with the regulatory evidence for each: type I and type II IFN signaling and T helper 1 (Th1) differentiation, B-cell receptor signaling and follicular homing, NF-κB negative regulation, plasma-cell biology and autophagy, and microRNA (miRNA)-mediated feedback [5,11]. One locus locates the defect precisely. At *OAS1*, the splice-site variant rs10774671 was associated with SjD in a meta-analysis of two European-ancestry cohorts (*p* = 2.59 × 10^−9^; odds ratio 0.75, 95% confidence interval [CI] 0.66–0.86). The risk allele shifts splicing from the p46 isoform toward p42, p48, and p44, which lack a translational response to type I IFN, placing the defect in interferon responsiveness rather than production [12]. Large-scale genome-wide association studies (GWAS) continue to add loci spanning T-cell receptor (TCR) signaling, autophagy, and Janus kinase–signal transducer and activator of transcription (JAK-STAT) signaling [5,13], so genetic risk rests on a multilayered architecture rather than a single defective pathway.

### 2.2. Functional Fine-Mapping and Salivary Gland eQTLs

Assigning each GWAS locus to its nearest gene has clear limits in SjD, because many risk variants sit in regulatory regions whose function depends on which element in which cell type they connect to. SjD risk loci are enriched in the enhancers of T helper, T regulatory, dendritic, and B cells, with Th1 enhancer enrichment the strongest signal [5]. With the recurrent detection of *STAT4* and *IL12A* and strong IFN-γ-related signaling in salivary gland lesions [14], this places genetic susceptibility on a multicellular immune axis spanning antigen sensing, a Th1-skewed cytokine response, B-cell activation, and failure of regulatory T (Treg) cell-mediated control.

More decisive is the finding that some risk variants act as expression quantitative trait loci (eQTLs) within salivary gland tissue: rs7749323 in the *TNFAIP3* region is an *IFNGR1* eQTL, two variants in *PRDM1-ATG5* link to *ATG5* eQTLs, and candidate variants at *TYK2* were proposed as minor salivary gland eQTLs for *ICAM5* and *EIF3G* [5]. The same allele can therefore act on cytokine or NF-κB signaling in immune cells while affecting IFN-receptor expression or autophagy regulation in the gland. Genetic risk surfaces in the target tissue, not in immune cells alone. This dovetails with reports that the salivary epithelium actively participates in inflammation, showing increased IFN-γ- and JAK/STAT-regulated gene expression with changes in tissue-remodeling genes such as *LTF*, *BMP3*, and *MMP7* [14]. Because salivary epithelial cells can build a local environment for B-cell activation through cytokines, chemokines, and B-cell-activating factor (BAFF), the target tissue sustains rather than merely receives the inflammatory response [4,15]. The reported eQTLs are, however, bulk-tissue-based, and the gland is heterogeneous, so bulk analysis cannot resolve which cell an expression change arises from. These results stand as candidate regulatory links requiring verification by single-cell eQTL analysis, single-cell RNA sequencing (scRNA-seq), and spatial transcriptomics.

### 2.3. Epigenetic Regulation and the X Chromosome

The type I IFN signature in SjD is persistent, with interferon-stimulated gene expression consistently elevated in salivary glands and peripheral blood, which a transient inflammatory response alone cannot explain. Hypomethylation of IFN-regulated genes such as *MX1*, *IFI44L*, and *PARP9* is consistently reported in whole blood and CD19^+^ B cells, and *OAS2* hypomethylation is confirmed in the minor salivary gland [16]. Coincident hypomethylation and increased expression at IFN-related genes suggests that the genetic predisposition can be maintained, beyond any single stimulus, as an IFN-prone state fixed epigenetically [17]. This signature associates with anti-Ro/SSA and anti-La/SSB status, indicating that antigen presentation, autoantibody formation, and IFN-centered epigenetic regulation join into one continuous host response [18]. Genetic predisposition lays the groundwork for the IFN state. The epigenetic layer fixes and sustains it.

Non-coding RNA forms a regulatory layer of the same kind. The inclusion of *PTTG1-MIR146A* among GWAS risk regions places miRNA-mediated regulation within this architecture [5]. miR-146a is a negative-feedback regulator of Toll-like receptor (TLR) and NF-κB signaling, so variation here offers a candidate mechanism for innate activation that fails to terminate. At the long non-coding RNA (lncRNA) level, *LINC01871*, induced by type II IFN, was proposed to link calcineurin/nuclear factor of activated T cells (NFAT) and TCR signaling to the differentiation and maintenance of cytotoxic T cells [19]. The non-coding transcriptome can therefore sustain chronic inflammation in an IFN-rich environment rather than simply mark it.

X-chromosome regulation offers a clue to the sex bias of SjD, in which women outnumber men roughly nine to one [20]. Sex hormones alone cannot explain this, and X-linked immune genes, X-inactivation skewing, and X-inactivation escape are thought to act together. X-linked gene-expression skewing was observed in the mesenchymal stromal cells of SjD salivary glands. This compartment is the non-epithelial, non-hematopoietic interstitium of fibroblasts, mesenchymal stromal cells, pericytes, and endothelium, and it organizes glandular inflammation rather than merely supporting it (Section 5.2). Reduced HLA-locus-derived miR-6891-5p was proposed to link to dysregulated *XIST* control and skewed X-linked silencing [18,21]. These are early observations from few tissues and cannot yet explain the female predominance, but a link between the HLA region and X-linked regulation would extend genetic risk into sex-biased epigenetic regulation and suggest that the salivary stromal compartment mediates a sex-specific host response.

**Table 1:** Genetic risk loci in Sjögren’s disease and their implicated immune pathways.

Risk Locus	Ancestry	Implicated Immune Pathway	Functional and Regulatory Evidence	Refs.
HLA class II (*HLA-DRB1*, *HLA-DQA1*, *HLA-DQB1*)	European, East Asian	Antigen presentation to CD4^+^ T cells	Strongest and most consistently replicated signal; HLA-DR expression in salivary gland epithelium adjacent to lymphocytic infiltrates	[5,8,10]
*IRF5-TNPO3*	European	Type I IFN response	Regulatory variants across the risk region; *IRF5* a candidate effector gene	[5,11]
*STAT4*	European, East Asian	Type I and II IFN, Th1 differentiation	Established locus; SjD risk loci show their strongest enrichment in Th1 enhancers	[5,11]
*IL12A*	European	IL-12 signaling and Th1 differentiation	Regulatory variants across the risk region; *IL12A* a candidate effector gene	[5,11]
*BLK* (*FAM167A-BLK*)	European	B-cell receptor signaling	Established locus; *BLK* encodes a B-cell receptor signaling kinase	[5,11]
*CXCR5* (*DDX6-CXCR5*)	European	B-cell and Tfh follicular homing	Established locus; *CXCR5* directs lymphocyte migration and lymphoid organization	[5,11]
*OAS1* (rs10774671)	European	Type I IFN effector response	Splice-acceptor variant shifting *OAS1* splicing from the p46 isoform to p42, p48 and p44, which lack a translational response to type I IFN	[12]
*TNIP1*	European	NF-κB negative regulation	Established locus acting in the negative regulation of NF-κB signaling	[11]
*TNFAIP3*	European, East Asian	NF-κB and IFN-related regulation	rs7749323 is an *IFNGR1* eQTL in bulk salivary gland tissue	[5,8]
*PRDM1-ATG5*	European	Plasma cell biology and autophagy	rs526531 and rs533733 are *ATG5* eQTLs in bulk minor salivary gland	[5]
*TYK2*	European	JAK-STAT cytokine signaling	Candidate *ICAM5* and *EIF3G* eQTLs in bulk minor salivary gland	[5]
*XKR6*	European	Not established	rs11250099 has a predicted regulatory link to *MTMR9* and is an *MTMR9* eQTL in T and B cells	[5]
*PTTG1-MIR146A*	European	miRNA-mediated TLR and NF-κB feedback	*MIR146A* a candidate effector gene in TLR and NF-κB negative feedback	[5]
*GTF2I* and *GTF2IRD1* (7q11.23)	East Asian	Not established	Causal gene and mechanism in SjD not established	[8]
*NCF1* (p.Arg90His)	European, East Asian	Phagocyte NADPH oxidase and ROS	p.Arg90His lowers NADPH oxidase-derived ROS; risk allele most frequent in East Asian populations	[22]
Thirteen new loci from trans-ancestry meta-analysis *	European and East Asian	Multiple immune pathways, largely shared with other autoimmune diseases	Meta-analysis of 10,003 cases: *TNFSF4*, *IKBKE*, *IDUA-SLC26A1*, *IKZF1*, *NCF1*, *HIP1*, *CLDN23-ERI1*, *ARID5B*, *PHRF1-IRF7*, *OVOL1*, *SH2B3*, *IRF8*, *TMEM86B*	[13]

* Reported as a conference abstract. eQTL, expression quantitative trait locus; HLA, human leukocyte antigen; IFN, interferon; ROS, reactive oxygen species; SjD, Sjögren’s disease; Tfh, T follicular helper; TLR, Toll-like receptor.

### 2.4. Trans-Ancestry Gaps and East Asian Under-Representation

HLA and non-HLA loci, salivary gland eQTLs, and epigenetic regulation are largely skewed toward European-ancestry cohorts, and it remains unclear which parts of the genetic architecture are conserved across ancestries. The *GTF2I*/*GTF2IRD1* region at 7q11.23 from a Han Chinese GWAS was interpreted as relatively characteristic of East Asians [8], although *NCF1* lies in the same region and has since been proposed as the causal gene [22]. Its missense variant p.Arg90His alters the p47phox subunit of phagocyte nicotinamide adenine dinucleotide phosphate (NADPH) oxidase, carries a high risk-allele frequency in East Asian populations, and associates with several autoimmune diseases including SjD with comparable effect sizes in Chinese and European American cohorts [22]. Meanwhile the reproducibility of *STAT4*, *TNFAIP3*, and the major histocompatibility complex (MHC) region indicates that core pathways are at least partly conserved, even as ancestry-comparison GWAS report non-negligible heterogeneity in risk-allele frequencies, effect sizes, and HLA haplotype structure [23].

That picture is changing. A GWAS and polygenic risk score analysis in a Taiwan Han cohort showed that large-scale SjD genetics is achievable in East Asia and that SjD shares part of its genetic basis with rheumatoid arthritis and systemic lupus erythematosus (SLE) [24], and a trans-ancestry meta-analysis of 10,003 European and East Asian cases reported thirteen new genome-wide significant loci including *NCF1* [13]. Studies focused on Korean SjD, by contrast, center on small whole-exome sequencing-based association studies [25], which cover the non-coding regulatory regions central to SjD poorly. Because HLA effects, autoantibody subtype distribution, and microbiome composition can all differ with ancestry and living environment, including diverse ancestry groups is a methodological necessity for testing the external validity of any mechanistic model, not a matter of inclusivity. The next task is to test, through functional genomics, how risk variants translate into a cell-type-specific host response in the salivary gland. Genetic and epigenetic architecture defines an IFN-prone predisposition but does not specify what triggers it, and the environmental interface most examined of late is the microbiome.

## 3. The Microbiome in Sjögren’s Disease: Function over Taxonomy

### 3.1. The Oral and Salivary Microbiome

The central sites for microbiome study in SjD are the oral cavity and the salivary glands (Table 2). Reduced secretion weakens pH buffering, antimicrobial peptide supply, mechanical washing, and mucin-mediated lubrication, so whether an oral microbiome change is intrinsic to the disease or a consequence of reduced saliva is hard to judge unless sampling site and salivary flow are considered together [26]. In pSS, the oral microbiota differed from a comparator group in which 10 of 25 participants had drug-related sicca, and an SjD-specific difference remained after stratifying by dryness and excluding taxa correlated with unstimulated whole salivary flow rate [27]. The same study showed that *Prevotella melaninogenica* invaded a salivary ductal epithelial cell line in vitro and induced MHC class I/II, CD80, and interferon-λ expression, and 16S in situ hybridization of archival labial gland sections detected bacteria at ductal cells and inflammatory foci [27]. These observations came from separate, non-overlapping sample sets, so the spatial closeness of oral dysbiosis and immune-activated epithelium was inferred rather than observed in the same patients.

Sampling compartment matters as much as sampling site, and here SjD data are thinnest. Whole saliva mixes microbiota from tongue, gingiva, buccal mucosa, and tooth surfaces, whereas parotid duct saliva approaches the ductal environment. Collected directly, composition differed significantly between anti-SSA-positive and anti-SSA-negative pSS, and diversity and *Firmicutes* abundance fell where sialectasis was present [35]. No study has yet profiled the salivary gland tissue microbiome and the saliva microbiome in the same SjD patients, so the relation between compartments must be inferred from comparable settings. Three are informative. In sialolithiasis, paired analysis of saliva and salivary stones from the same patients found high diversity in saliva but low diversity and evenness in the stone, and although some taxa were shared, the compositions differed significantly [47]. This is direct evidence that an intraductal compartment is not read off from saliva. In IgG4-related disease, which like SjD presents as chronic lymphoplasmacytic sialadenitis, the saliva microbiome formed a cluster distinct from SjD, indicating compartment signals that a shared sicca phenotype does not explain [48]. In inflammatory bowel disease, where the comparison has been made at scale, fecal microbiota differed clearly from both rectal swab and tissue microbiota, with swabs closer to but still distinct from tissue [49]. Together these imply that whole saliva is a poor surrogate for the intraductal and intraglandular niche where the epithelial responses of interest occur. Two methodological requirements follow: tissue and intraductal samples are low-biomass, so reagent and laboratory contamination can dominate the recovered profile unless negative controls are sequenced alongside and reported [50], and sequence-based detection in tissue should be corroborated orthogonally, as by the in situ hybridization above [27].

Functional clues are accumulating. *Haemophilus parainfluenzae*, enriched with the largest effect size in healthy controls, increased programmed death-ligand 1 (PD-L1) expression in a salivary epithelial cell line and suppressed CD4^+^ T-cell proliferation [34], raising the possibility that oral dysbiosis involves loss of an immunoregulatory commensal and not only expansion of pathobionts. Taxon-level results, however, disagree across studies. Compositional abnormalities are repeatedly observed in saliva from patients with dry mouth and pSS, yet the direction of alpha-diversity change diverges, and some reports show no difference [30]. The core of oral microbiome change therefore lies in ecological instability, a shift toward an acidified and caries-prone ecology, and changes at the mucosal immune interface, more than in any single genus.

### 3.2. Gut Dysbiosis and Depletion of SCFA-Producing Bacteria

**Figure 2:**
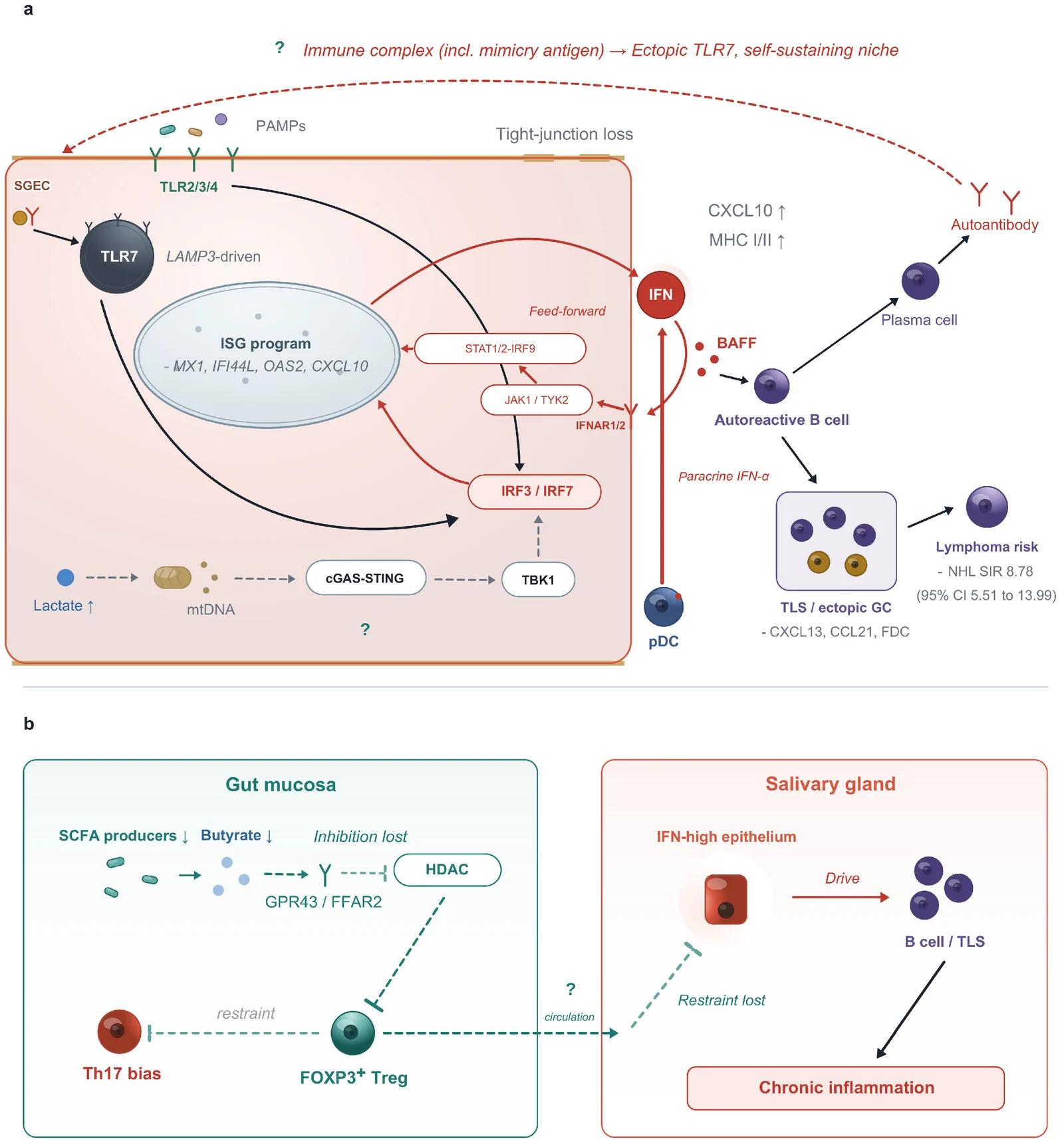
Microbial input converging on epithelial type I interferon (IFN) amplification, with the short-chain fatty acid (SCFA) brake weakened in a separate compartment. (**a**) Pattern-recognition input and type I IFN amplification at the salivary gland epithelial cell (SGEC). Solid arrows denote links supported by more than one study. Dashed arrows rest on single studies and are marked with a question mark: molecular mimicry between the Vientovirus capsid and Ro52/SSA, extended here to α-carbonic anhydrases with carbonic anhydrase 6 (CA6) and parotid secretory protein (PSP); the lactate to mitochondrial DNA (mtDNA) to cyclic GMP-AMP synthase and stimulator of interferon genes (cGAS-STING) route; and the immune-complex route into ectopic Toll-like receptor 7 (TLR7), which this review proposes on the basis of the reported LAMP3-TLR7 loop [41,53,54,55,56,57,58,59]. (**b**) Weakened SCFA restraint in the gut mucosa and its proposed transmission to the salivary gland, where a question mark marks the cross-compartment step, which has not been measured. The conditions under which each proposed step would be rejected are given in Section 6.2. T-bars denote inhibition, and in (**b**) dashed lines denote signaling and inhibition that are weakened or lost. Colors encode axis and cell lineage: the type I IFN axis and pro-inflammatory cells in red, the SCFA restraint axis in teal, and B-lineage cells in purple; gold cells within the TLS are T cells, and the TLR7 endosome is shown in slate. Abbreviations: BAFF, B-cell-activating factor; CI, confidence interval; FDC, follicular dendritic cell; FFAR2, free fatty acid receptor 2; GC, germinal center; GPR43, G-protein-coupled receptor 43; HDAC, histone deacetylase; IFNAR, type I interferon receptor; IRF, interferon regulatory factor; ISG, interferon-stimulated gene; JAK1, Janus kinase 1; LAMP3, lysosome-associated membrane protein 3; MHC, major histocompatibility complex; NHL, non-Hodgkin lymphoma; PAMP, pathogen-associated molecular pattern; pDC, plasmacytoid dendritic cell; SIR, standardized incidence ratio; STAT, signal transducer and activator of transcription; TBK1, TANK-binding kinase 1; Th17, T helper 17; TLR, Toll-like receptor; TLS, tertiary lymphoid structure; Treg, regulatory T cell; TYK2, tyrosine kinase 2.

The central message of gut microbiome research is the functional decline of short-chain fatty acid (SCFA)-producing bacteria, not which taxa rose or fell (Figure 2b). Acetate, propionate, and butyrate are produced by fiber-fermenting bacteria and are linked to epithelial barrier maintenance, mucosal tolerance, and regulatory T-cell-related immune regulation [51,52]. Reading dysbiosis functionally avoids the inconsistency of taxonomic lists while extracting the immunological implications shared across SjD.

Reduced mucosal microbiome diversity accompanied ocular and systemic disease severity in patients and mouse models [28]. In 42 pSS patients and 35 controls, severe dysbiosis was more frequent in pSS (21% versus 3%, *p* = 0.018) and was associated with higher disease activity on the EULAR Sjögren’s Syndrome Disease Activity Index (ESSDAI; 13 versus 5, *p* = 0.049), lower C4 (0.11 versus 0.17 g/L, *p* = 0.004), and higher fecal calprotectin (110 versus 33 μg/g, *p* = 0.001) [29]. Gut dysbiosis has also been associated with dry-eye severity [32]. Direct evidence of functional change is growing. In treatment-naive pSS, altered functional potential and five candidate microbial peptides mimicking pSS-related autoepitopes were reported [39], and gut microbiota was associated with systemic inflammation, intestinal permeability, and blood FOXP3 expression [33]. Whole-metagenome sequencing of feces from 206 pSS patients and 355 controls, integrated with serum and fecal metabolomes in a paired subset of 75 patients and 66 controls, reported multi-kingdom changes across 49 bacterial, 19 fungal, and 1323 viral species with metabolome remodeling and presented reduced SCFA as a functional indicator beyond taxonomy [42]. A pSS-SLE comparison showed that the two share much of their gut dysbiosis while differing more clearly in oral microbiota [7], supporting the view that gut dysbiosis is a mucosal disturbance shared with systemic autoimmunity, whereas oral and salivary dysbiosis is tied more closely to glandular dysfunction.

### 3.3. Hyposalivation as a Confounder

The most important confounder in SjD microbiome research is hyposalivation, because saliva nourishes oral microbes and handles clearance, so the oral microbiota can change when saliva declines even without an SjD-specific immune mechanism [26]. The studies pressing this hardest hold that salivary flow explains microbiome variation better than disease label. Whole-saliva microbiota were similar between pSS and non-SjD sicca [26,31], hyposalivation associated more closely with dysbiosis than did SjD diagnosis in a female cohort [37], and site-specific oral signatures were associated with reduced glandular function [38]. Not all oral dysbiosis is so explained. A parotid saliva study found disease status and anti-Ro/SSA positivity associated with microbiome variation independently [35], and even within a mild-xerostomia group, *H. parainfluenzae* abundance differed between pSS and non-SjD sicca [34]. Separating disease from secretion signals therefore requires a non-SjD sicca comparator matched for reduced secretion, oral health, and medication, with site-specific sampling and population-specific references.

### 3.4. Evidence for Causality from Mendelian Randomization and Treatment

Evidence that can directly claim causality is limited. Two-sample Mendelian randomization studies based on MiBioGen GWAS have suggested potential causal associations between gut microbiota and SjD but converge on the existence of a signal while diverging on which taxa they implicate. With overlapping European exposure data, weak instruments, and limited taxonomic resolution (Table 2), they remain hypothesis-generating [45,46].

Treatment-reversal signals call for similar care. Profiling gut, oral, and vaginal microbiomes in 133 pSS patients, 56 non-pSS patients, and 40 controls found that after hydroxychloroquine (HCQ), the dysbiosis partly recovered, while composition remained disturbed [36]. Whether HCQ normalized the microbiome directly or altered the inflammatory environment and induced secondary ecological change is hard to disentangle, so partial reversal indicates that the microbiome responds to the disease state, not that it causes the disease. Animal models give clues to directionality. Germ-free humanized mice receiving SjD patient feces showed reduced CD4^+^Foxp3^+^ Treg and desiccation-induced corneal barrier damage [43], and non-obese diabetic (NOD) mice orally colonized with *H. parainfluenzae* showed eased focal sialadenitis, improved salivary function, fewer IFN-γ^+^ T cells in draining lymph nodes, and restored salivary diversity [44]. These support the hypothesis that loss of a commensal, or colonization with one, can affect pathology, without confirming causation in humans. Because hyposalivation and oral ecological factors act as major confounders, the microbiome remains at the stage of observation, leaving host–microbe crosstalk a partly supported hypothesis.

## 4. Microbe-to-Host Crosstalk

### 4.1. Molecular Mimicry and the Antigenic Repertoire

Molecular mimicry holds that when a microbial antigen resembles a host autoantigen structurally, a cross-reactive response can spread to the autoantigen. Under genetic susceptibility and type I IFN dominance, such recognition is triggered more readily, and once cross-reactivity is established, the response can widen through epitope spreading. Ro52, a principal SjD autoantigen, is an E3 ubiquitin ligase also known as TRIM21 that negatively regulates type I IFN production by degrading IFN regulatory factors such as IRF3 [60], so autoimmunity against Ro52 borders the type I IFN loop directly and differs functionally from responses to the RNA-binding autoantigen Ro60/SSA.

Direct evidence that a resident microbe can supply such an antigen comes from Ro60. Orthologs of human Ro60 are carried by skin, oral, and gut commensals, are bound by patient anti-Ro60 autoantibodies, and activate Ro60-reactive patient T cells, and germ-free mice monocolonized with a Ro60-ortholog-bearing commensal developed anti-Ro60 responses and glomerular immune-complex deposition [61]. That work was in lupus, so it establishes the route rather than the SjD-specific case. A single-center study using shotgun metagenomics of saliva from 35 SjD patients and 25 controls reported that a Vientovirus capsid protein showed sequence similarity to Ro52/SSA and antibody cross-reactivity and that mice immunized with the capsid peptide acquired anti-Ro52/SSA antibodies and SjD-like features [41]. Without independent replication it should be treated as one emerging mechanism.

The SjD autoantibody repertoire extends beyond Ro/SSA and La/SSB, and the newer tissue-specific autoantibodies matter here because their targets are salivary proteins at the host–microbe interface. Antibodies to salivary gland protein 1 (SP-1), carbonic anhydrase 6 (CA6), and parotid secretory protein (PSP) were identified in two animal models and appeared earlier than anti-Ro or anti-La. In patients, they were found in 45% of those meeting SjD criteria who lacked anti-Ro and anti-La, and among patients with idiopathic xerostomia and xerophthalmia of under two years’ duration, 76% had anti-SP-1 and/or anti-CA6, while only 31% had anti-Ro or anti-La [53]. In registry sera stratified by focus score, anti-CA6 was significantly more frequent in the focus score 0 group than in groups with focal sialadenitis (*p* = 0.032 and *p* = 0.006), while anti-Ro and anti-La marked the more severely affected glands [54]. Their diagnostic specificity is unresolved, which is why they were not adopted into the 2016 classification criteria. In an idiopathic dry eye practice without systemic disease, 60% of patients expressed one of the three, twice the proportion expressing anti-Ro or anti-La [55], and the registry analysis rested on only nine focus score 0 sera. They are therefore best read not as classification markers but as evidence about where the earliest autoimmune targeting occurs.

Read that way, two of the three targets bear directly on a host–microbe model. CA6 is a secreted α-carbonic anhydrase of saliva, and α-carbonic anhydrases are widely distributed among bacteria. Homology between human carbonic anhydrase II and the α-carbonic anhydrase of *Helicobacter pylori*, with the homologous segments containing an HLA-DRB1 binding motif, is the basis of a long-standing mimicry proposal in autoimmune pancreatitis [56]. Anti-CA6 is thus a mimicry candidate whose host target is itself a salivary secretory protein, making it more specific to the glandular interface than Ro60 or Ro52. PSP, now designated BPIFA2, belongs to the bactericidal/permeability-increasing (BPI)-fold family of innate-defense proteins, binds lipopolysaccharide, and acts as the principal surfactant of saliva. Mice lacking BPIFA2 have saliva with the surface tension of water, carry less salivary lipopolysaccharide, and show mild metabolic endotoxemia [57]. Autoimmunity against PSP is therefore autoimmunity against a component of salivary antimicrobial defense, connecting the autoantibody repertoire to the barrier failure of Section 4.4 rather than to antigen presentation alone. Both connections are hypotheses, and Section 6.2 states the observation that would refute the CA6 proposal. More broadly, mimicry is one of several routes supplying antigenic stimulation and does not by itself explain chronic tissue inflammation. It acquires disease-related significance only when amplified by the pattern recognition receptor (PRR) and type I IFN axis.

### 4.2. Pattern Recognition Receptors and the Type I IFN Amplifier

The PRR and type I IFN axis is the amplifier converting sporadic antigenic stimulation into a persistent circuit of tissue inflammation (Figure 2). In SjD salivary tissue and peripheral blood, a consistent rise in interferon-stimulated gene (ISG) expression has been repeatedly observed [17,62]. This signature links genetic susceptibility, epithelial stress, microbial sensing, and autoantibody generation into one self-reinforcing loop, converting the genetically defined IFN predisposition into tissue-level activity.

Type I IFN production is triggered by nucleic-acid-sensing PRRs: endosomal TLR3 (double-stranded RNA, dsRNA), TLR7 (single-stranded RNA, ssRNA) and TLR9 (CpG DNA), and the cytosolic cyclic GMP-AMP synthase and stimulator of interferon genes (cGAS-STING) pathway. These are not confined to professional immune cells. Salivary gland epithelial cells (SGECs) from SjD patients functionally express TLR2, TLR3, and TLR4 and respond to dsRNA [63]. The high-lactate environment of the SjD gland was proposed to activate cGAS-STING through mitochondrial DNA damage and leakage [58], and ectopic lysosome-associated membrane protein 3 (LAMP3) expression was reported to drive ectopic TLR7 expression in SGECs, forming a positive feedback loop amplifying the type I IFN response [59]. The target tissue is therefore itself an active participant in IFN production, while plasmacytoid dendritic cells (pDCs) recruited to the gland activate the IFN pathway and, through immune-complex-driven endosomal TLR stimulation, raise type I IFN activity systemically [64,65].

Downstream effects include upregulation of MHC classes I and II and induction of chemokines such as CXCL10 and of BAFF. SGECs secrete BAFF upon IFN stimulation. In patient monocytes, a type I IFN signature was detected in 55% of pSS cases versus 4.5% of controls, and signature-positive patients showed higher BAFF mRNA [15,66]. Because BAFF promotes survival and clonal expansion of autoreactive B cells, the IFN circuit leads directly to adaptive immune activation, and in experimental models, the STING and type I IFN axis drove interaction between marginal zone B cells and T follicular helper cells [67]. Microbial dysbiosis is thus reinterpreted as a change in PRR-ligand load and epithelial stress rather than in taxa alone. What matters is how strongly a change stimulates the host’s nucleic-acid- and microbe-sensing systems. Once the circuit forms, nucleic-acid-containing immune complexes repeatedly stimulate endosomal TLRs, so it sustains itself even if the initial microbial trigger disappears. We propose that this self-sustaining property is central to the chronicity of SjD inflammation.

Two qualifications belong with this circuit. Not every interferon signal implicated in SjD is type I, and the evidence sits at different levels. Risk-locus enrichment is strongest in Th1 enhancers, a genomic annotation rather than a tissue measurement [5]; minor salivary gland epithelium shows prominent IFN-γ-related signaling [14]; *LINC01871* is type II IFN-induced [19]; and the epithelial response to *P. melaninogenica* was reported as interferon-λ [27], whereas specifically type I evidence includes the blood and salivary ISG signature, the LAMP3-TLR7 loop, and the lactate to cGAS-STING route [58,59,62,65,66]. Second, an elevated ISG score records exposure to interferon, not its cellular source, so it cannot separate epithelium that amplifies interferon from epithelium responding to interferon produced by infiltrating pDCs. Describing the epithelium as an amplifier is therefore a mechanistic proposal, and resolving type I from type II and type III activity within the same gland is one of the measurements this review argues is missing.

### 4.3. Microbial Metabolites and Loss of Immune Restraint

If the PRR and IFN axis switches inflammation on, dysfunction of the microbial-metabolite pathway leaves it uncontrolled (Figure 2b). The two work like accelerator and brake. The fall in butyrate following SCFA-producer decline has direct consequences for immune regulation, because butyrate acts as a histone deacetylase inhibitor, raising histone acetylation at the *Foxp3* promoter and promoting Foxp3^+^ Treg differentiation [51,52]. That relationship is established mainly in colonic Treg models. Butyrate has, however, been given to the NOD mouse, the prototype SjD model, where it raised salivary flow and reduced glandular inflammation through IL-10-producing B cells rather than Treg induction [68], so reduced SCFA can weaken peripheral tolerance by more than one route, and the balance between them in human SjD is unresolved. Reduced Treg and corneal barrier damage in humanized mice receiving patient feces are animal-level clues that such regulatory failure connects to the SjD phenotype [43]. Causality is unproven in human SjD, and reduced SCFA production is entangled with disease activity, diet, medication, and oral intake. Rather than a sole cause, this pathway lowers the threshold of systemic immunity and lets the PRR and type I IFN loop persist once formed.

### 4.4. Epithelial Barrier Dysfunction and Direct Glandular Infection

The epithelial barrier is the interface at which antigenic stimulation, type I IFN amplification, and weakened immune regulation are physically realized. Because normal saliva maintains barrier homeostasis through antimicrobial peptides, IgA, mucin, and buffering capacity, hyposalivation weakens this defense and promotes local dysbiosis and epithelial stress, making reduced saliva one arm of a vicious cycle and not only a confounder for microbiome data. At the molecular level, IL-17 from infiltrating lymphocytes reduced tight junction proteins including occludin in SGECs through NF-κB [69], and a single study proposed that loss of the tricellular tight junction protein tricellulin contributes, through the JAK/STAT1/miR-145 axis, to macromolecular leakage and hyposalivation [70]. Tight junction breakdown raises epithelial permeability and immune exposure to microbial products, autoantigens, and damage-associated molecular patterns, which epithelial cells relay to immune cells through antigen presentation and chemokine secretion. Damaged epithelium is therefore the tissue-level foundation on which the PRR and IFN circuits operate, consistent with the classical concept of autoimmune epithelitis [2].

This framework predicts that events damaging the glandular epithelium directly should also register as risk, and the epidemiology is consistent. In a systematic review and meta-analysis of 12 studies and 158,539 participants, a history of infection was associated with pSS with a pooled odds ratio of 2.73 (95% CI 1.93–3.86), among the larger environmental effect sizes reported [71]. Sialadenitis is the most direct form this exposure takes. Among 668 pSS patients, 242 (36.2%) had experienced parotid gland swelling. Of these, 78 (32.2%) had swelling before the SjD diagnosis was established and a further 86 (35.5%) at diagnosis, so glandular inflammatory episodes commonly precede or accompany recognition of the disease [72]. The pediatric presentation makes the point more sharply. Among 20 children presenting with recurrent parotitis, 10% met adult American-European Consensus Group criteria, and 40% met proposed pediatric criteria for SjD [73]. This is compatible with an ascending ductal route in which oral organisms reach the ductal epithelium, as bacteria detected at ductal cells and inflammatory foci in labial gland sections suggest [27], and initiate the epithelial stress and PRR signaling described above, while the resulting secretory loss further degrades clearance.

Two cautions constrain this. Protopathic bias is the dominant alternative. Undiagnosed early SjD may itself present as sialadenitis, so an association between prior sialadenitis and later diagnosis may record delayed recognition rather than causation. The meta-analytic infection estimate is also assembled from heterogeneous exposure definitions and relies largely on retrospective ascertainment [71]. Distinguishing the two requires a design the available studies have not used: a population-scale cohort treating sialadenitis as a time-varying exposure, and excluding episodes that fall within a defined lag window before diagnosis, so that only episodes clearly antedating disease contribute. Microbial signals alter the host-cell state, and the altered tissue reshapes the microbial niche in turn, and this bidirectional reinforcement sets the stage for chronic inflammation, lymphocyte accumulation, and ultimately B-cell activation and tertiary lymphoid structure (TLS) formation.

### 4.5. B-Cell Activation, Tertiary Lymphoid Structures, and Lymphoma Risk

The mechanisms converge on chronic B-cell activation. SjD is characterized by B-cell hyperactivity with serological abnormalities including autoantibodies, rheumatoid factor, hypergammaglobulinemia, hypocomplementemia, and cryoglobulinemia, the last two recognized as predictors of poor prognosis [74]. At the center lies the IFN and BAFF axis: BAFF supports survival and selection of autoreactive B cells, epithelial and stromal cells create a chemokine-rich microenvironment drawing lymphocytes into the tissue, and the immune complexes generated stimulate PRRs again, so the gland becomes a self-sufficient immune niche. The structured form of this inflammation is the TLS, comprising B cells, T cells, and a follicular dendritic cell-like network organized progressively by lymphoid chemokines such as CXCL13 and CCL21 into an ectopic germinal center-like structure [75,76,77]. In patients whose salivary biopsy showed such structures, non-Hodgkin lymphoma incidence was markedly higher than in those without (about 14% versus 0.8%) [78], and at meta-analysis, the risk is elevated relative to the general population with a pooled standardized incidence ratio of 8.78 (95% CI 5.51 to 13.99) [79]. This risk is the clinical consequence of long-term epithelial-immune interaction and should not be read as direct evidence of a particular microbiome change or of mimicry. The mechanisms to this point are proposed at tissue and pathway resolution. Which cell and which state they occur in can be answered only at single-cell and spatial resolution.

## 5. Multi-Omics Evidence in Human Tissue and Body Fluids

### 5.1. Salivary Gland scRNA-Seq and Pathogenic Cell States

**Figure 3:**
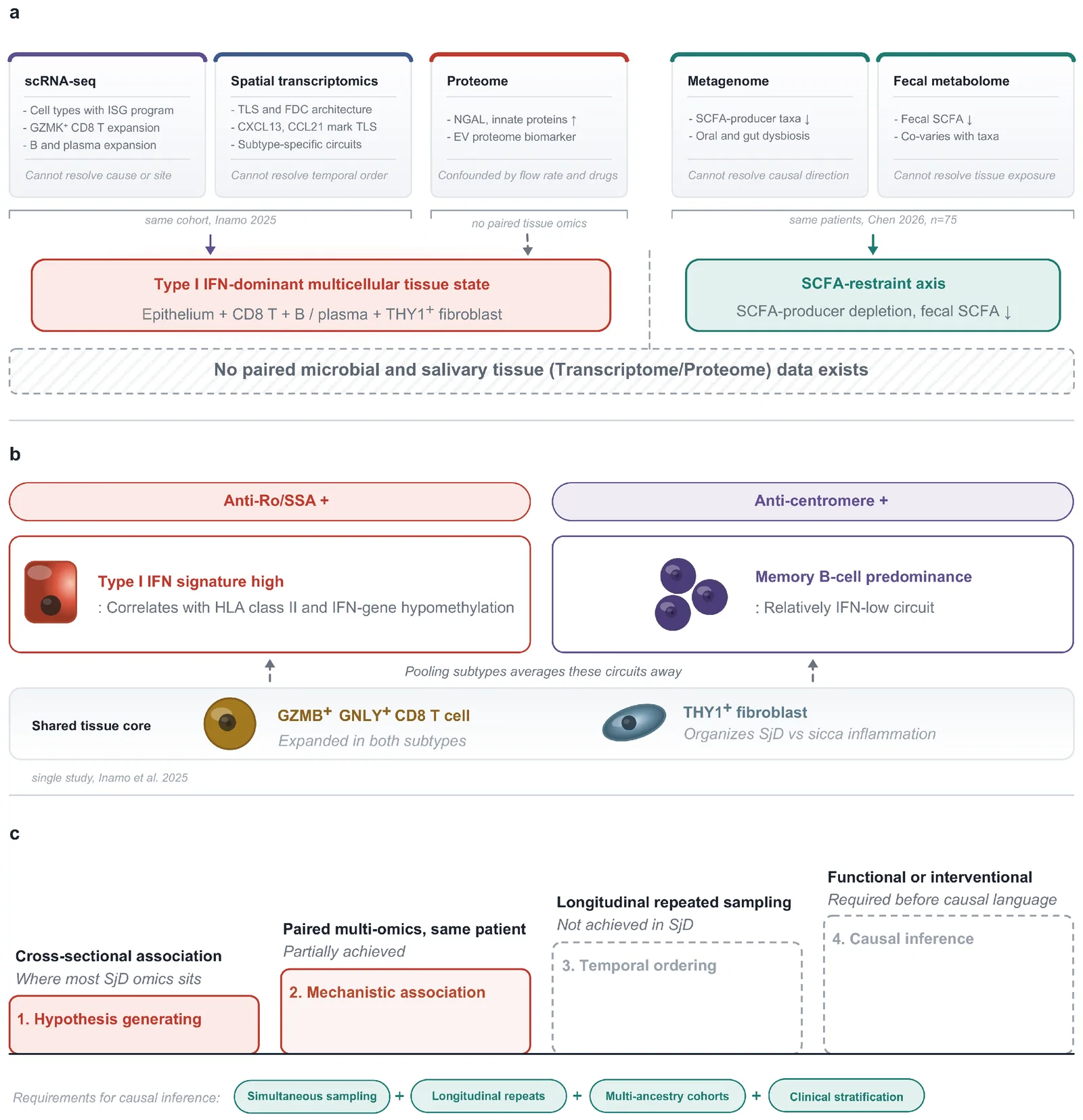
Convergence of each omics layer on one tissue state or one microbial axis, with the two convergences coming from different patients. (**a**) Five omics layers grouped by cohort, each annotated with the inference it cannot support on its own. The gut metagenome and the fecal metabolome were measured in the same patients [42]. The scRNA-seq and spatial transcriptomics data come from one cohort without stated donor overlap [82]. The salivary proteome comes from a separate cohort, so no paired microbial and salivary tissue dataset exists. (**b**) Autoantibody-subtype differences within the same tissue state, from a single study [82]. (**c**) Levels of evidence for human study designs in SjD. Solid boxes mark the levels SjD omics has reached, dashed boxes those it has not, and the row beneath lists the design features that causal inference would require, to which Section 6.2 adds compartment-resolved sampling. In (**a**), solid arrows denote convergence within a cohort, and the dashed arrow from the proteome denotes evidence from a separate cohort not yet integrated; upward and downward arrows denote increase and decrease in SjD relative to controls. Colors encode axis and cell lineage: the type I IFN axis and IFN-high epithelium in red, the SCFA restraint axis in teal, B and plasma cells in purple, cytotoxic CD8 T cells in gold, and THY1^+^ fibroblasts in slate. Abbreviations: EV, extracellular vesicle; FDC, follicular dendritic cell; GNLY, granulysin; GZMB, granzyme B; GZMK, granzyme K; HLA, human leukocyte antigen; IFN, interferon; ISG, interferon-stimulated gene; NGAL, neutrophil gelatinase-associated lipocalin; SCFA, short-chain fatty acid; scRNA-seq, single-cell RNA sequencing; SjD, Sjögren’s disease; THY1, Thy-1 cell surface antigen; TLS, tertiary lymphoid structure.

The tissue response in SjD appears as an emerging pathogenic cell state in which several cell types are remodeled together (Figure 3). The bulk transcriptome captured the type I IFN signature only as a whole-tissue average. scRNA-seq resolves it to the cellular level. Peripheral-blood scRNA-seq showed expansion of cytotoxic CD4^+^ T lymphocytes and increased *TCL1A* expression in B cells [80]. In the labial gland, tissue-resident cytotoxic CD8^+^ subsets including granzyme K^+^ CD8^+^ T cells were identified in proportion to inflammation [81], and in a single-cell and spatial study, IFN-response genes were upregulated across several cell subtypes rather than in any one cell [82].

Here the epithelial cell emerges as an active participant in antigen presentation, chemokine secretion, and the IFN response rather than a passively destroyed target [4,14], although for the reason given in Section 4.2, these data place the epithelium inside the tissue IFN response without establishing it as the amplifier. B- and plasma-cell expansion is associated with autoantibody generation and TLS formation, cytotoxic CD8^+^ states are tied to epithelial damage, and fibroblast and endothelial changes remodel the microenvironment so inflammatory cells enter and settle. Because scRNA-seq is cross-sectional, it cannot prove causal relationships between cell states. Increased IFN-responsive epithelial cells may reflect a response to an already-formed inflammatory microenvironment rather than its initiation, and biopsy location, focus score, treatment status, and degree of tissue damage also affect cell composition.

### 5.2. Spatial Transcriptomics and Autoantibody Subtypes

Spatial transcriptomics interprets cell states by position, since the same B or T cell means something different around a duct, around an epithelial lesion, or inside or at the margin of a TLS. Characterizing the SjD salivary gland TLS at molecular and spatial levels showed that it matures locally as a dynamic response, with CXCL13 and CCL21 confined to sites of TLS formation and stromal cells and a follicular dendritic cell-like network acting as key elements of local B-cell activation and antibody production [83].

The association between autoantibody subtype and tissue architecture is an important clue (Figure 3b). Comparing anti-SSA-positive and anti-centromere-positive glands by scRNA-seq, TCR, and B-cell receptor sequencing and spatial transcriptomics, granzyme B^+^ granulysin^+^ CD8^+^ cytotoxic T cells were the core, most expanded population regardless of autoantibody status, while cytokine signaling diverged by subtype: an activated interferon signature in anti-SSA-positive glands versus enhanced transforming growth factor-β (TGF-β) signaling and a more prominent memory B-cell compartment in anti-centromere-positive glands. THY1^+^ fibroblasts expressing complement genes and chemokines were identified as a key stromal hub organizing inflammation in SjD relative to sicca [82]. Autoantibody subtype can therefore reflect differences in tissue-level cell arrangement and inflammatory circuitry beyond a serological marker, and pooling patients into one group can mask subtype-specific signals. Spatial proximity alone, however, cannot establish the direction or causality of signaling. These require functional experiments or longitudinal studies.

### 5.3. Proteome, Metabolome, and the Cross-Omics Integration Gap

The proteome and metabolome test whether transcriptome-level cell states translate into protein and metabolite change. Liquid chromatography-mass spectrometry of saliva confirmed upregulation of inflammatory and innate-immune proteins including neutrophil gelatinase-associated lipocalin, and an extracellular vesicle-derived proteome was proposed as a promising biomarker [84]. Because saliva is strongly affected by flow rate, oral hygiene, medication, and infection, these changes must be read against the epithelial IFN response, barrier change, and B-cell activation rather than listed as individual biomarkers, and a strong transcriptome signal unconfirmed at protein level warrants caution. The metabolome adds metabolic reprogramming. Increased lactate has been proposed as a functional indicator of a shift to aerobic glycolysis in activated immune and epithelial cells, and in a cell model accumulated lactate and damaged mitochondrial DNA activated cGAS-STING and induced a type I IFN response [58], while joint analysis of gut microbiome and fecal metabolome showed compositional change associated with metabolome remodeling including SCFA [42].

The largest gap in SjD research is not a shortage of individual omics data but the absence of any study measuring different pathophysiological layers together in the same patients (Figure 3a). Within a layer, same-patient integration has been achieved: gut metagenome and fecal metabolome in a paired subset of 75 pSS patients [42] and salivary gland scRNA-seq and spatial transcriptomics in one cohort, though on different participant numbers and without stated donor overlap [82]. This does not carry across layers. The design has itself been attempted. A pilot measured the epithelial transcriptome and ocular-surface microbiome at the same conjunctival site in 11 SjD and four non-SjD participants and integrated these with blood transcriptome and gut microbiome in the same participants [40], but because it used a conjunctival swab and no salivary gland biopsy, the glandular tissue layer alone remains unpaired. Closing the gap is therefore not impossible in principle. The tissue-acquisition barrier can be overcome using cohorts already undergoing diagnostic minor labial salivary gland biopsy. The aim is not to gather data in one place but to ask, within the same patients, whether microbial compositional change tracks a particular metabolite change, whether that change appears together with the epithelial IFN state, TLS maturation, B-cell subset expansion, and salivary protein change, and how those relate to autoantibody status, complement reduction, cryoglobulinemia, and lymphoma risk. Only such data can separate microbiome change as an accompanying phenomenon, as a regulator of the inflammatory circuit, or as a therapeutic target in particular patient groups.

## 6. Synthesis and a Falsifiable Research Agenda

### 6.1. What Is Supported and What Remains Open

Four concepts are supported relatively consistently (Figure 1): a central interferon-driven circuit [66,85]; a target tissue that is the stage of an autoimmune epithelitis rather than passively damaged [2,4]; B-cell hyperactivity and TLS maturation associated with severity and lymphoma risk [74,78]; and repeatedly reported oral and gut dysbiosis with SCFA-producer depletion [7]. The core message is not that the microbiome causes SjD but that these belong with genetic susceptibility in one host–microbe framework to be tested with same-patient integrated omics.

Three questions remain open. The reproducibility of molecular mimicry is untested. Mimicry between Vientovirus and Ro52/SSA shows that a microbial antigen can connect to anti-Ro/SSA autoimmunity but rests on a single-center study [41], and antigenic similarity alone does not sustain chronic inflammation without IFN dominance and BAFF signaling. Whether dysbiosis is cause or consequence is unclear, because hyposalivation confounds oral dysbiosis, while disease-associated signals persist in designs matched for reduced secretion [26,35]. The cross-omics gap of Section 5.3 remains, which is why the proposed cascade stays a partly supported model. These uncertainties are connected within one process, so they need stepwise testing with designs matched to each hypothesis.

### 6.2. Directions for Integrative Validation

Future research should analyze microbes, cell states, proteins, metabolites, and clinical measures together in the same patient, across diverse ancestries, and with longitudinal repeat sampling. Sampling should be as close to simultaneous as possible, with microbiome, salivary gland transcriptome, and body-fluid proteome and metabolome collected at one time point and compartment-resolved, since saliva is a poor surrogate for the intraductal and intraglandular niche (Section 3.1), and low-biomass tissue sampling requires sequenced negative controls. Repeat sampling before and after treatment and across changes in disease activity would establish whether microbial change precedes tissue inflammation, moves with it, or follows progression. Because treating patients as one homogeneous group dilutes the differences seen in autoantibody subtypes, TLS maturation, and lymphoma high-risk groups, clinically stratified analysis should run in parallel. Changes should be interpreted by pathway, namely PRR-ligand load, reduced SCFA, lactate change, epithelial barrier change, IFN and BAFF signaling, and the B-cell and TLS axis, rather than by a single bacterium or gene set, and levels of evidence must be distinguished (Figure 3c): single association analysis generates hypotheses, same-patient paired multi-omics assesses mechanistic association, and longitudinal repeat sampling establishes temporal order.

For the model to function as a hypothesis rather than a narrative, falsification conditions must accompany it. It yields three falsifiable predictions. First, in a same-patient design, depletion of SCFA-producing bacteria should covary with the epithelial ISG score independently of dietary fiber intake, medication, and disease activity. If the association disappears after adjustment, the proposal that the SCFA brake regulates the epithelial type I IFN state is rejected. Second, in a longitudinal design, change at the microbial layer should precede the IFN-high tissue state. If it instead follows, the proposed direction of causality is overturned, and microbiome change must be reinterpreted as a consequence. Third, if anti-CA6 reflects mimicry at the glandular interface (Section 4.1), anti-CA6-positive patients should carry a higher relative abundance of α-carbonic-anhydrase-encoding taxa in the salivary compartment than anti-CA6-negative patients matched for salivary flow. If abundance is unrelated to anti-CA6 status, that route is rejected, and the antibody must be read as a marker of glandular injury alone. Only with such explicit conditions does this model stand as a testable hypothesis.

## 7. Mechanism-Targeted Therapy

Clinical trials read the same axis from the other end, because every mechanism-targeted agent reaching the clinic blocks one identifiable node of it (Table 3). Historically, there was no US Food and Drug Administration (FDA)- or European Medicines Agency (EMA)-approved disease-modifying therapy for SjD, and approvals were limited to muscarinic agonists for dry symptoms [86]. On 8 June 2026, China’s National Medical Products Administration (NMPA) approved telitacicept, the first systemic disease-modifying therapy approved for SjD. Figure 1b maps these target classes onto the disease phase at which each has been tested.

Table 3 sets out the trial, phase, endpoint, and development status for each agent. Three patterns in that table carry the argument. First, the axis of BAFF and a proliferation-inducing ligand (APRIL) now has both positive phase 3 evidence and an approval. Ianalumab met its primary endpoint with monthly dosing in two phase 3 studies [87], and telitacicept significantly improved the 24-week ESSDAI over placebo in a Chinese phase 3 study of anti-SSA-positive patients [88]. Yet the absolute effect is modest. Ianalumab’s pooled ESSDAI change was −6.5 versus −5.3 for placebo, a difference of −1.2, so modulating a single axis cannot control the disease as a whole. Second, targets adjacent to that axis are advancing on the same logic: dazodalibep against CD40L [89] and iscalimab against CD40 [90] gave positive phase 2 results from opposite sides of one costimulatory pair, and nipocalimab against the neonatal Fc receptor lowered Clinical ESSDAI (ClinESSDAI) and serum IgG by blocking IgG recycling [91], identifying autoantibody-mediated activity as a target downstream of the IFN circuit without correcting epithelial barrier or microbiome abnormalities. Neither the CD40-CD40L axis nor the effector principle is secure, however. Novartis discontinued iscalimab in SjD in January 2025 on benefit-risk grounds despite the positive result, and rituximab failed its primary endpoint in major randomized trials despite a clear B-cell-targeting mechanism [92,93]. That gap between a clear mechanism and a negative outcome suggests that response depends on patient group, tissue lesion, autoantibody subtype, and TLS maturation and that the existence of a target does not by itself guarantee efficacy.

The third pattern is the most consequential for the axis this review follows. Every SjD target that has reached clinical success or regulatory approval belongs to the downstream B-cell effector axis. In the upstream innate and type I IFN axis, three programs reached the clinic, and none reached therapeutic establishment. Hydroxychloroquine, acting partly by blocking endosomal TLR7 and TLR9 signaling, did not separate from the placebo in the randomized JOQUER trial (17.9% versus 17.2%; *p* = 0.98) [94], yet a substudy of the same trial showed it lowered the type I IFN score and ISG expression while clinical response did not improve [95], and it shifted the microbiome without normalizing it (Section 3.4). The drug therefore moved two intermediate readouts of the upstream axis, and neither shift translated into symptom relief. Adezkibart (AMG 329), an anti-FMS-like tyrosine kinase 3 ligand (FLT3L) antibody depleting dendritic and plasmacytoid dendritic cells, met a futility threshold in phase 2 and was discontinued in 2026 [96], and the oral STING inhibitor ASP5502 was terminated in 2026 for strategic reasons at phase 1 [97]. These outcomes differ in nature, but none established the upstream axis.

**Table 3:** Mechanism-targeted therapies in Sjögren’s disease: clinical evidence and development status.

Axis	Target and Mechanism	Agent(s) [Ref.]	Trial, Phase, *n*, Registration	Primary Endpoint	Primary Result	Population	Development Status
**Adaptive immune and B-cell effector axis**	TACI-Fc fusion protein blocking BAFF and APRIL	Telitacicept [88]	Phase 3; *n* = 381; NCT05673993	ESSDAI change from baseline at week 24	Week 24 LSM change −4.4 (160 mg) and −3.0 (80 mg) versus −0.6 for placebo; both *p* < 0.0001	Anti-Ro/SSA-positive adults with ESSDAI ≥ 5; 79 centers in China	Primary endpoint met; approved by China’s National Medical Products Administration on 8 June 2026; global phase 3 UPSTREAM SjD ongoing *
	Anti-BAFF receptor antibody with B-cell-depleting activity	Ianalumab [87]	Phase 3 NEPTUNUS-1 (NCT05350072) and NEPTUNUS-2 (NCT05349214); *n* = 779 pooled	ESSDAI change from baseline at week 48	Monthly dosing met the primary endpoint in both trials; the every-3-months arm of NEPTUNUS-2 did not (difference −0.5; *p* = 0.34). Pooled monthly versus placebo, ESSDAI change −6.5 versus −5.3 (*p* = 0.0031)	Adults with active SjD	Primary endpoint met for monthly dosing; US FDA Breakthrough Therapy Designation in January 2026; no SjD approval reported *
	CD40L (dazodalibep) and CD40 (iscalimab) costimulation blockade	Dazodalibep [89]; iscalimab [90]	Dazodalibep: phase 2, population 1 *n* = 74, NCT04129164. Iscalimab: phase 2b TWINSS, cohort 1 *n* = 173, NCT03905525	Dazodalibep: ESSDAI change at day 169. Iscalimab: dose-response in ESSDAI change at week 24	Dazodalibep −6.3 versus −4.1 for placebo (LSM difference −2.2; *p* = 0.0167). Iscalimab: significant dose-response, placebo-adjusted LSM −3.0 (150 mg) and −2.9 (600 mg)	Dazodalibep population 1: ESSDAI ≥ 5. Iscalimab cohort 1: ESSDAI ≥ 5 and ESSPRI ≥ 5	Both phase 2 trials positive; dazodalibep phase 3 ongoing; iscalimab TWINSS completed, but the SjD program was discontinued in January 2025 on benefit-risk grounds
	Anti-FcRn antibody	Nipocalimab [91]	Phase 2 DAHLIAS; *n* = 163; NCT04968912	ClinESSDAI change from baseline at week 24	Placebo-adjusted LSM difference −2.65 for 15 mg/kg (90% CI −4.03 to −1.28; *p* = 0.0018), with reduced serum IgG	Adults with moderate to severe SjD, positive for anti-Ro60 or anti-Ro52 IgG	Phase 2 positive; US FDA Breakthrough Therapy Designation in November 2024; phase 3 DAFFODIL ongoing
	Anti-CD20 antibody	Rituximab [92,93]	TEARS: phase 2 and 3, *n* = 120, NCT00740948. TRACTISS: phase 3, *n* = 133, ISRCTN65360827	TEARS: improvement ≥ 30 mm in 2 of 4 VAS at week 24. TRACTISS: ≥30% reduction in oral dryness or fatigue VAS at week 48	Neither TEARS nor TRACTISS met its primary endpoint	TEARS: recent-onset pSS scoring ≥ 50 mm on 2 of 4 VAS. TRACTISS: pSS with fatigue and oral dryness	Pivotal SjD trials negative; no SjD approval
**Innate immune and type I IFN axis**	Endosomal TLR7 and TLR9 signaling blockade	Hydroxychloroquine [94,95]	JOQUER; randomized, double-blind, placebo-controlled; *n* = 120 (56 hydroxychloroquine, 64 placebo); NCT00632866	Proportion with a ≥30% reduction between weeks 0 and 24 in 2 of 3 numeric analog scales for dryness, pain and fatigue	17.9% versus 17.2% for placebo (odds ratio 1.01, 95% CI 0.37 to 2.78; *p* = 0.98)	Adults with primary SjD; 15 university hospitals in France	Primary endpoint not met; a substudy of the same trial found a reduced type I IFN score and lower ISG expression without clinical improvement
	Anti-FLT3L antibody depleting dendritic and plasmacytoid dendritic cells	Adezkibart (AMG 329) [96]	Phase 2; *n* = 209; NCT06312020	Co-primary: ESSDAI change at week 48 in population 1 and ESSPRI change at week 24 in population 2	No efficacy result reported; stopped after meeting a prespecified interim futility criterion	Two SjD populations: systemic disease activity and high symptom burden	Discontinued for futility; insufficient efficacy rather than a safety concern
	Oral STING inhibitor	ASP5502 [97]	Phase 1 first-in-human, single and multiple ascending dose; *n* = 116; NCT06544642	Safety, tolerability, and pharmacokinetics	No result reported; terminated for a stated company strategic decision	Healthy adults and adults with primary SjD	Terminated on 5 March 2026; no efficacy conclusion available

* Phase 3 results reported as a conference abstract. Development status is current to July 2026. APRIL, a proliferation-inducing ligand; BAFF, B-cell-activating factor; CI, confidence interval; ClinESSDAI and ESSDAI, (Clinical) EULAR Sjögren’s Syndrome Disease Activity Index; ESSPRI, EULAR Sjögren’s Syndrome Patient Reported Index; FcRn, neonatal Fc receptor; FLT3L, FMS-like tyrosine kinase 3 ligand; IFN, interferon; ISG, interferon-stimulated gene; LSM, least squares mean; SjD, Sjögren’s disease; STING, stimulator of interferon genes; TACI, transmembrane activator and CAML interactor; TLR, Toll-like receptor; VAS, visual analogue scale.

## 8. Conclusions

SjD is being reinterpreted from a disease defined solely by immune-cell abnormality into a host-response disease in which host genetic and epigenetic susceptibility, the target-tissue epithelium, and the microbial environment operate together. This review proposes that reinterpretation as a single continuous host–microbe axis: HLA and non-HLA susceptibility and an IFN-prone epigenetic state form the foundation, on which oral and gut dysbiosis and SCFA-producer depletion are layered. Microbial signals are translated into the epithelial PRR and type I IFN amplification circuit, leading to BAFF-dependent B-cell activation, ectopic germinal-center formation, and increased non-Hodgkin lymphoma risk. Two axes run through this flow: a type I IFN accelerator and a weakened SCFA brake.

The model is not yet a causal chain established in human SjD, and its value lies not in the conclusion but in the testable hypothesis, the three falsification conditions of Section 6.2, and the verification design they imply: integrated studies measuring the microbiome, salivary gland scRNA-seq and spatial transcriptomics, proteome, and metabolome with clinical measures in the same patients, followed by longitudinal and interventional designs establishing the direction and time axis of causality. Therapeutically, the efficacy of BAFF-APRIL-B-cell effector strategies is supported by the approval of telitacicept and the phase 3 results of ianalumab, but modulating a single axis cannot control the disease as a whole, and programs aimed at the upstream innate and type I IFN axis have not reached therapeutic establishment. As integrated same-patient evidence accumulates, it will become possible to advance toward a precise therapeutic strategy that distinguishes which layer of intervention is meaningful in which patients.

## Figures and Tables

**Table 2:** Microbiome studies in Sjögren’s disease: design, sampling site, and key findings.

Study [Ref.]	Method	Cohort (Site, *n*)	Design	Key Finding	Confounder Control and Caveats
Human studies: 16S rRNA gene-targeted profiling					
de Paiva 2016 [28]	16S rRNA sequencing; desiccating stress mouse model	Conjunctiva: SjD 15, HC 8, rosacea 6; tongue: SjD 10, HC 11; stool: SjD 10, Human Microbiome Project controls 45	Cross-sectional and experimental	Lower fecal diversity with greater disease severity; antibiotic-induced dysbiosis worsened ocular disease in mice	No multivariable adjustment; stool controls taken from a separate dataset
Mandl 2017 [29]	GA-map Dysbiosis Test (targeted 16S rRNA probe assay)	Stool; pSS 42, HC 35	Cross-sectional	Severe dysbiosis in 21% of pSS versus 3% of controls, with higher ESSDAI, lower complement C4, and higher calprotectin	Controls matched for age and sex
van der Meulen 2018 [26]	16S rRNA sequencing	Oral washings; pSS 36, non-SjD sicca 85, HC 14	Cross-sectional	Salivary secretion explained 9% of compositional variation versus 5% for disease status; lower *Streptococcus* was flow-independent	Adjusted for age, dental status and smoking; salivary flow modeled separately
Rusthen 2019 [30]	16S rRNA sequencing	Saliva; pSS 15, non-SjD sicca 15, HC 15	Cross-sectional	pSS and non-SjD sicca both differed from HC, and pSS also differed from non-SjD sicca; alpha diversity was similar across groups	Non-SjD sicca and healthy comparators
van der Meulen 2019 [7]	16S rRNA sequencing	Stool and saliva; pSS 39, SLE 30, population controls 965	Cross-sectional, two diseases	Gut dysbiosis shared between pSS and SLE, whereas oral profiles differed	Adjusted for age, sex, body mass index, proton pump inhibitor use, and sequencing depth
Sembler-Møller 2019 [31]	16S rRNA sequencing	Whole saliva; pSS 24, non-SjD sicca 34	Cross-sectional	Salivary composition and diversity comparable between pSS and non-SjD sicca	Non-SjD sicca comparator; salivary flow and oral health considered
Alam 2020 [27]	16S rRNA sequencing; cell culture; 16S in situ hybridization	Oral mouthwash 50 (pSS 25, control 25 including 10 drug-related sicca); archival labial salivary gland 23 (SjD 15, non-SjD 8); non-overlapping sets	Cross-sectional and experimental	Disease-associated profiles in both the dry and the non-dry stratum; *Prevotella melaninogenica* induced MHC class I and II, CD80, and interferon-λ in gland epithelium; bacteria found in gland tissue	Stratified by dryness; human, cell and tissue sample sets are separate
Moon 2020 [32]	16S rRNA sequencing	Stool; SjD 10, dry eye controls 14, HC 12	Cross-sectional	Gut composition was associated with dry eye signs and severity	Adjusted for age, sex, study group, and hydroxychloroquine use
Cano-Ortiz 2020 [33]	16S rRNA sequencing	Stool; pSS 19, HC 19	Cross-sectional	Gut differences were associated with systemic inflammation, intestinal permeability and blood *FOXP3* expression	Controls matched for age, sex, and body mass index
Tseng 2021 [34]	16S rRNA sequencing; salivary gland epithelial cell model (A253)	Saliva; pSS 8, HC 16; separate pSS versus non-SjD sicca comparison stratified by xerostomia	Cross-sectional and experimental	*Haemophilus parainfluenzae*, most enriched in HC, raised epithelial PD-L1 and reduced CD4^+^ T-cell proliferation	Stratified by xerostomia; small saliva cohort
Kim 2022 [35]	16S rRNA sequencing	Parotid duct saliva; SjD 23, non-SjD sicca 9	Cross-sectional	Composition differed by anti-Ro/SSA status; lower diversity and lower *Firmicutes* with sialectasis	Non-SjD sicca comparator; saliva collected directly from the parotid duct
Wang 2022 [36]	16S rRNA sequencing	Gut, oral and vaginal; pSS 133, non-pSS 56, HC 40; 56 of the pSS group resampled 3 to 6 and 6 to 12 months after starting hydroxychloroquine	Cross-sectional and uncontrolled longitudinal	Richness and composition shifted after hydroxychloroquine but stayed different from HC	Follow-up within the same individuals; no randomized untreated comparison
Saúco 2023 [37]	16S rRNA sequencing	Saliva; SjD 25, non-SjD 25; women only	Cross-sectional	Oral dysbiosis associated more with hyposalivation than with the SjD diagnosis	Women only, comparable age
Kamounah 2025 [38]	16S rRNA sequencing	Four oral sites; pSS 23, non-SjD sicca 36, HC 21	Cross-sectional	Differences were site-specific and associated mainly with salivary gland hypofunction	Non-pSS sicca and healthy comparators; sampling resolved by site
Human studies: metagenomics and paired multi-omics					
Jia 2023 [39]	Shotgun metagenomics	Stool; treatment-naive pSS 78, HC 78, SLE 49	Cross-sectional	Compositional and functional differences, including candidate molecular mimicry peptides	Treatment-naive participants; matched HC and an SLE comparator
Nguyen 2025 [40]	Blood and conjunctival RNA sequencing with ocular surface and gut microbiome profiling	Peripheral blood, conjunctival swab and rectal swab; SjD 11, non-SjD 4	Cross-sectional pilot	Type I interferon and complement pathways upregulated in blood; gut enriched for *Lactobacillus reuteri*	Very small pilot; microbiome and host transcriptome paired in the same participants, but at the conjunctiva and without salivary gland tissue
Zhang 2025 [41]	Shotgun metagenomics with viral sequence analysis	Saliva; SjD 35, HC 25	Cross-sectional and experimental	Expanded *Siphoviridae* bacteriophages and increased eukaryotic viral sequences including *Vientovirus*; a Vientovirus capsid protein showed sequence similarity and antibody cross-reactivity to Ro52/SSA, and immunized mice acquired anti-Ro52/SSA antibodies	Single center, not yet independently replicated; healthy comparator only
Chen 2026 [42]	Shotgun metagenomics with serum and fecal metabolomics	Stool; pSS 206, non-pSS 355. Paired serum and fecal metabolome; pSS 75, non-pSS 66	Cross-sectional	Multi-kingdom alterations across 49 bacterial, 19 fungal and 1323 viral species, with reduced short-chain fatty acids	Large non-pSS comparator; metabolome measured in a paired subset
Experimental models					
Schaefer 2022 [43]	Fecal microbiota transfer into germ-free mice	Human stool donors: SjD 7, dry eye 4, HC 20; germ-free recipient mice	Experimental	SjD-associated microbiota lowered CD4^+^Foxp3^+^ regulatory T cells and worsened corneal barrier damage	Defined donor colonization; small SjD donor group
Tseng 2025 [44]	Salivary quantitative PCR; oral colonization of non-obese diabetic (NOD) mice	Saliva; SjD 30, HC 30. NOD mice 22, 11 per group	Cross-sectional and experimental	Oral *Haemophilus parainfluenzae* lowered focus score, improved salivary flow and reduced interferon-γ-positive T cells	Controlled colonization; single mouse strain
Genetic causal inference					
Cao 2023 [45]; Wang 2024 [46]	Two-sample Mendelian randomization	Exposure: MiBioGen, 18,340 participants from 24 cohorts. Outcome, FinnGen: 2247 cases and 332,115 controls [45]; 1981 cases and 300,162 controls [46]	Instrumental variable analysis	Possible causal associations, but signals were weak and implicated different taxa	Valid only if the instrumental variable assumptions hold; predominantly European data and a shared exposure dataset

ESSDAI, EULAR Sjögren’s Syndrome Disease Activity Index; HC, healthy control; MHC, major histocompatibility complex; pSS, primary Sjögren’s syndrome, used where the cited study enrolled only that group; SjD, Sjögren’s disease; SLE, systemic lupus erythematosus.

## Data Availability

No new data were created or analyzed in this study. Data sharing is not applicable to this article.

## Abbreviations

APRIL, a proliferation-inducing ligand; BAFF, B-cell-activating factor; BPI, bactericidal/permeability-increasing; BPIFA2, BPI fold containing family A member 2; CA6, carbonic anhydrase 6; cGAS, cyclic GMP-AMP synthase; CI, confidence interval; ClinESSDAI, Clinical EULAR Sjögren’s Syndrome Disease Activity Index; dsRNA, double-stranded RNA; EMA, European Medicines Agency; eQTL, expression quantitative trait locus; ESSDAI, EULAR Sjögren’s Syndrome Disease Activity Index; ESSPRI, EULAR Sjögren’s Syndrome Patient Reported Index; EV, extracellular vesicle; FcRn, neonatal Fc receptor; FDA, US Food and Drug Administration; FDC, follicular dendritic cell; FLT3L, FMS-like tyrosine kinase 3 ligand; GC, germinal center; GWAS, genome-wide association study; HCQ, hydroxychloroquine; HDAC, histone deacetylase; HLA, human leukocyte antigen; IFN, interferon; ISG, interferon-stimulated gene; JAK-STAT, Janus kinase–signal transducer and activator of transcription; LAMP3, lysosome-associated membrane protein 3; lncRNA, long non-coding RNA; MHC, major histocompatibility complex; miRNA, microRNA; NFAT, nuclear factor of activated T cells; NF-κB, nuclear factor kappa B; NHL, non-Hodgkin lymphoma; NMPA, National Medical Products Administration; NOD, non-obese diabetic; PAMP, pathogen-associated molecular pattern; pDC, plasmacytoid dendritic cell; PD-L1, programmed death-ligand 1; PRR, pattern recognition receptor; PSP, parotid secretory protein; pSS, primary Sjögren’s syndrome; SCFA, short-chain fatty acid; scRNA-seq, single-cell RNA sequencing; SGEC, salivary gland epithelial cell; SIR, standardized incidence ratio; SjD, Sjögren’s disease; SLE, systemic lupus erythematosus; SP-1, salivary gland protein 1; STING, stimulator of interferon genes; TACI, transmembrane activator and CAML interactor; TCR, T-cell receptor; TGF-β, transforming growth factor-β; Th1 and Th17, T helper 1 and T helper 17; TLR, Toll-like receptor; TLS, tertiary lymphoid structure; Treg, regulatory T cell. Stromal cells of the salivary gland are referred to throughout as the non-epithelial, non-hematopoietic interstitial compartment of the gland, comprising fibroblasts, mesenchymal stromal cells, pericytes and endothelial cells.
